# Biomimetic Vision for Zoom Object Detection Based on Improved Vertical Grid Number YOLO Algorithm

**DOI:** 10.3389/fbioe.2022.905583

**Published:** 2022-05-20

**Authors:** Xinyi Shen, Guolong Shi, Huan Ren, Wu Zhang

**Affiliations:** ^1^ School of Information and Computer, Anhui Agricultural University, Hefei, China; ^2^ School of Electrical Engineering and Automation, Wuhan University, Wuhan, China

**Keywords:** bionic vision, zoom target detection, deep learning, image segmentation, simple linear iterative clustering, light/dark co-occurrence scene

## Abstract

With the development of bionic computer vision for images processing, researchers have easily obtained high-resolution zoom sensing images. The development of drones equipped with high-definition cameras has greatly increased the sample size and image segmentation and target detection are important links during the process of image information. As biomimetic remote sensing images are usually prone to blur distortion and distortion in the imaging, transmission and processing stages, this paper improves the vertical grid number of the YOLO algorithm. Firstly, the light and shade of a high-resolution zoom sensing image were abstracted, and the grey-level cooccurrence matrix extracted feature parameters to quantitatively describe the texture characteristics of the zoom sensing image. The Simple Linear Iterative Clustering (SLIC) superpixel segmentation method was used to achieve the segmentation of light/dark scenes, and the saliency area was obtained. Secondly, a high-resolution zoom sensing image model for segmenting light and dark scenes was established to made the dataset meet the recognition standard. Due to the refraction of the light passing through the lens and other factors, the difference of the contour boundary light and dark value between the target pixel and the background pixel would make it difficult to detect the target, and the pixels of the main part of the separated image would be sharper for edge detection. Thirdly, a YOLO algorithm with an improved vertical grid number was proposed to detect the target in real time on the processed superpixel image array. The adjusted aspect ratio of the target in the remote sensing image modified the number of vertical grids in the YOLO network structure by using 20 convolutional layers and five maximum aggregation layers, which was more accurately adapted to “short and coarse” of the identified object in the information density. Finally, through comparison with the improved algorithm and other mainstream algorithms in different environments, the test results on the aid dataset showed that in the target detection of high spatial resolution zoom sensing images, the algorithm in this paper showed higher accuracy than the YOLO algorithm and had real-time performance and detection accuracy.

## Introduction

Biomimetic remote sensing is an interdisciplinary discipline that covers a variety of technical disciplines, including computer technology, sensor technology, image processing technology and other technologies (Duan et al., 2021; Chen T. et al., 2021). Biomimetic remote sensing technology uses the electromagnetic wave reflection and radiation information of the target to capture and image the electromagnetic wave reflected and emitted by the electromagnetic wave sensor installed on the spacecraft, such as satellites and hot air balloons. It has become a comprehensive new technology to process and analyse the characteristic information of specific targets. An important application field of remote sensing technology is land resource monitoring, such as land use information statistics, land cover dynamic monitoring and data updates (Lee and Ke, 2018; Giri et al., 2019; Islam et al., 2021). With the development of spatial information science, remote sensing images are widely used and provide researchers with reliable data sources. The classification and location of specific targets in remote sensing images can be obtained through target recognition technology, which can be applied to species identification and classification, crop area estimation and monitoring, crop nutrient and water status monitoring and other fields. Therefore, the detection and recognition of remote sensing targets has important research value (Shadrin et al., 2020; Yu et al., 2021; Hu et al., 2015).

Currently, geographic information systems (GISs) have been widely used in many fields. However, there is a bottleneck problem in GIS applications, that is, how to quickly extract target information and update GIS data. In the field of photogrammetry and remote sensing, target extraction and recognition are technical hot spots that are urgently needed in production applications but still far from production applications and are one of the focuses of remote sensing (Li and Liu 2020). Therefore, object extraction of images is of great significance. The objects detected by remote sensing are divided into three categories: point targets, linear targets (such as roads, rivers, etc.) and planar targets (such as buildings, etc.) (Abdollahi et al., 2020; Huang et al., 2022). Rich texture information and spatial information are important characteristics of high spatial resolution zoom sensing images that distinguish them from other images (Huang et al., 2021). High-precision classification of high-spatial-resolution images plays an important role in agriculture, urban planning, environmental monitoring and other fields. How to extract the target effectively is always a difficult problem. At present, the most commonly used target detection methods are mainly target motion detection. The literature (Mahmoodi and Salajeghe, 2019; Zhang et al., 2019; Sun et al., 2020) has proposed the background difference method, interframe difference method, optical flow method, etc., but only moving targets can be detected, while stationary targets or slow-moving targets cannot be effectively detected, and targets cannot be accurately classified (Jiang et al., 2021). Thanks to deep learning, mainly convolutional neural networks and candidate region algorithms (Haut et al., 2018; Wang and Dou et al., 2019), a huge breakthrough in target detection has been made. The literature (Jin et al., 2020; Peng et al., 2020; Khan et al., 2021) defines RCNN, Fast RCNN, Faster-RCNN and YOLO, among which YOLO is a brand-new target detection method that integrates target determination and target recognition. End-to-end detection not only achieves fast detection but also achieves better performance (Sadykova et al., 2020; Liu X. et al., 2021).

To accurately detect targets, this paper first abstracted the light and shade of high-resolution zoom sensing images and adopts SLIC superpixel segmentation. Second, a YOLO algorithm with an improved number of vertical grids was proposed to detect targets in real time on the processed superpixel image array. YOLO grid framework could divide the image into different regions, so as to predict the boundary box and probability of each region. These boundary boxes would be weighted by the predicted probability. After adjusting the vertical mesh according to the structure of the curved zoom microlens array, the prediction accuracy of the algorithm would increase and the speed would also increase. The comparison with the improved algorithm in different environments showed that the proposed method had better accuracy. At the same time, compared with other mainstream target algorithms, it was verified that the improved algorithm in this paper was suitable for scenes requiring both speed and precision. The increasing resolution of remote sensing images had become a reality for realizing agricultural work from relatively macro large-scale species identification and monitoring to individual plant species identification and monitoring of the drop changes in crop nutrition, diseases and insect pests. The improvement of image resolution would improve the precision of future agricultural work.

## Related Work

In computer vision, images with medium and high accuracy have become the data types commonly used by researchers, especially in species identification and classification in agriculture, such as agricultural vegetation classification, land use classification, crop classification, tree species identification, etc., (Roslim et al., 2021; Chen Z. et al., 2021; Li et al., 2021; Yan et al., 2021). To solve the common problems of zoom sensing image segmentation algorithms, such as poor robustness, easy loss of edge information and narrow scope of application, the core task of zoom sensing image target detection is to judge whether there is a target in zoom sensing images and to detect, segment, extract and classify it.

Edges contain the most concentrated rich local image information, and the Gaussian filter used for smoothing also blurs the edges of the image. In order to more accurately examine the edges in image segmentation, Liu et al. (2019) chose guaranteed edge guide filtering features and used Canny operator and wave decomposition. Each band edge detection processing was conducted, and then the edges would be integrated into a result image. Compared with the traditional edge detection operator, the segmentation result is smoother, but for remote sensing images with complex background and changeable environment, the model generalization performance is poor and the detection effect is not good. Li et al. (2019) proposed the aircraft target detection model DC-DNN of remote sensing images based on a deep neural network. This model relied on a small number of image-level labels and eliminates overlapping frames and false detection frames through a detection frame suppression algorithm to complete pixel-level target segmentation and detection of zoom sensing images. The average pixel accuracy of the DC-DNN supervised FCN depth model in the three datasets was 81.47%, and the detection accuracy of aircraft targets in remote sensing images was 95.78%. Li et al. (2019) improved the Faster-RCNN network by adding the attention mechanism module into the feature extraction network to obtain more information about the target to be paid attention to and suppress other useless information to adapt to the problem of complex backgrounds and small targets caused by the large field of vision of remote sensing images. The result was 12.2% higher than that of the original Faster-RCNN. Alganci et al. (2020) compared and evaluated the performance of target detection algorithm based on deep learning convolutional neural network in aircraft target detection task in remote sensing image. Fast RCNN achieved the highest accuracy, but the detection speed was too slow to meet the needs of real-time detection. SSD algorithm had the lowest detection performance, and YOLOv3 achieved a balance between accuracy and detection speed. Hou and Jiang, (2021) improved YOLOv4’s trunk feature extraction network by using dense link network multiplexing features, strengthened the detection ability of small targets, and obtained a target detection algorithm more suitable for detecting aircraft in remote sensing images, which effectively optimized the problem of aircraft target detection in zoom sensing images by the YOLOv4 algorithm. However, targets with large occlusions in bionic vision system cannot be detected accurately. In general, compared with other algorithms, YOLO series algorithms can meet the requirements of real-time and high efficiency in zoom sensing image detection. Compared with YOLOv4, YOLOv5 had higher accuracy and no reduction in detection speed.

Because the YOLO algorithm has good comprehensive performance in detection speed and accuracy, it is applied to short-range target detection. However, it is difficult to adapt to the huge difference of target size and scale in target detection (Liu, Y. et al., 2021; Yun et al., 2022). According to the structure of curved zoom microlens array, the single-stage sub eye lens of bionic human eye imaging system is a microlens array with uneven focal length. Its transverse image points are in the plane of photoelectric receiver, while the longitudinal image points will have obvious defocus. Therefore, the detection effect become better after adjusting the vertical grid. Based on YOLO deep learning network and the characteristics of image distortion caused by the special imaging principle of bionic zoom vision system, a new real-time detection method of zoom sensing targets is proposed in this paper.

## Zoom Target Detection Bionic Vision Method

### Zoom Image Texture Feature Detection Index

**FIGURE 1:**
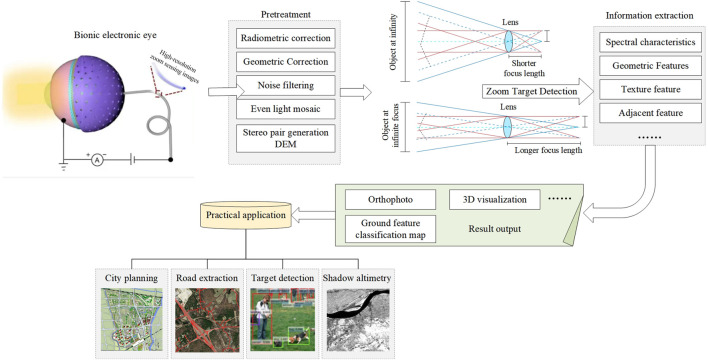
High-resolution zoom image processing and application.

The information of high-resolution images is diversified and complex. For such data sources, information processing technology cannot stay on several simple technologies. For the determination of the target area, in general, if the scope of the target is fixed or has access to the area of specific geographic coordinates, GPS and GIS technology can be used to guide the satellite to the specified area which is most intriguing, and fixed location monitoring of the data search can greatly improve the efficiency and precision of target detection (Wu et al., 2020; Yang et al., 2021; Zhao et al., 2022). The overall processing of zoom sensing images is shown in Figure 1. First, the high-resolution zoom sensing image is preprocessed, such as geometric correction and noise filtering. The generated image is extracted for information, usually spectral and texture features. The output results, such as ground feature classification maps, can be further used in practical application scenes, such as road extraction (Al-Masni et al., 2018; Li and He, 2020).

A Texture expresses the local properties of the image and reflects the relationship between pixels in the local area. It is generally believed that texture is a grey co-occurrence matrix composed of texture elements repeatedly arranged according to certain rules. It is defined as the probability of *n* grey at the *x*' point (*i*', *j*'), which is displaced *d* away from (*i*, *j*) in any direction, when the grey level at pixel (*i*, *j*) of point *x* in the image is *M*. The grey cooccurrence matrix can be used to extract feature parameters to describe the difference in ground object spatial features. Besides, the richness of texture information quantitatively describes the texture features of remote sensing images.

Different from image features such as gray and color, texture is represented by the gray distribution of pixels and their surrounding spatial neighborhood, which is represented as the local texture information. While various parameters of texture features reflect the properties of global features, they also describe the surface properties of the scene corresponding to the image or image region. The four features of images are entropy (ENT), HomoM (HOM), contrast (CON), and angular second moment (ASM). The synergy mainly reflects the degree of local variation of the image texture, that is, the uniformity of the image. Contrast can be used to reflect the clarity of the texture or the quality of the visual effect of the image. Information entropy can measure the richness of image texture information. The angle second moment is mainly used to observe the thickness of the image texture. The specific calculation formulas are as follows:

$$\mathrm{ENT}=-\sum_{i,j}p{\left(i,j\right)}^{2}\log_{2}p\left(i,j\right)$$ (1)

$$\mathrm{CON}=\sum_{i=0}^{L-1}\sum_{j=0}^{L-1}|i-j{|}^{2}p\left(i,j\right)$$ (2)

$$\mathrm{HOM}=\sum_{i,j}\frac{p\left(i,j\right)}{{\left(i-j\right)}^{2}+1}$$ (3)

$$\mathrm{ASM}=\sum_{i}\sum_{j}p{\left(i,j\right)}^{2}$$ (4)

Where *p* (*i*, *j*) is the element in the normalized grey cooccurrence matrix; *i* and *j* are the row and column numbers of pixel points; and *L* is the grey level. The distortion of biomimetic remote sensing images will damage their texture features, so we use texture features to detect the distortion of biomimetic remote sensing images.

### Bionic Sensing Image Correction Model

Due to the problem that remote sensing images cannot extract effective information caused by dark shadows, a TMF (truncated media filter) is applied which not only remove noise but also enhance the boundary. First, the pixels with the same characteristics in the dark part of the image are labelled, and the pixels of the labels are connected to form the dark part boundary. The image expression formed by the dark part pixels in the boundary region is as follows:

$$BndCon\left(T\right)=\left|\left\{q|q\in T,q\in Bnd\right\}\right|{\sqrt{\left|\left\{q|q\in T\right\}\right|}}^{-1}$$ (5)

**FIGURE 2:**
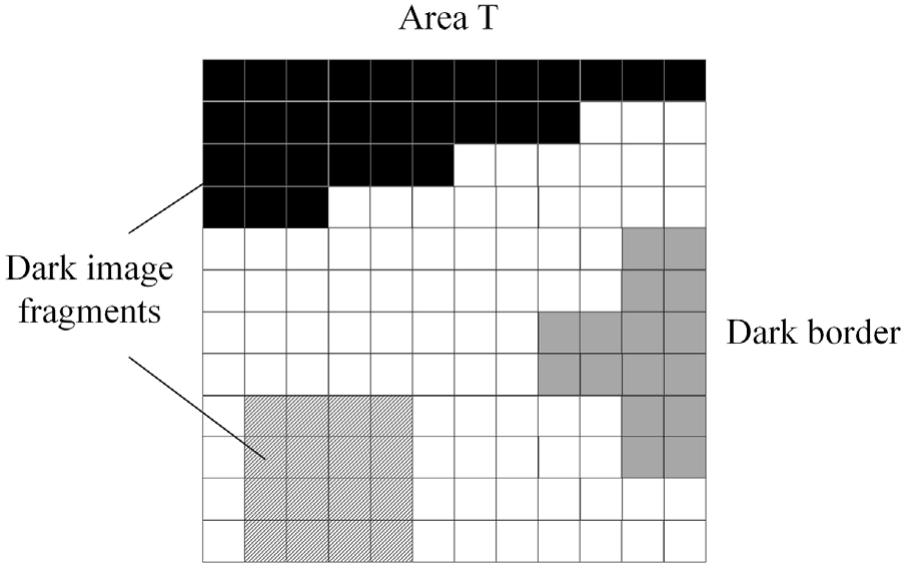
Truncated median filter model.

In the formula, *Bnd* is the collection of dark boundary pixels, and *q* is the image fragment of the dark region. The TMF model detects the target by analyzing the associated parameters of region *T* and image light and dark pixels, which can realize the rapid identification and separation of image between light and dark scenes. The TMF model is shown in Figure 2.

The SLIC super pixel segmentation method is adopted in the model (Kumar and Bhandari et al., 2021; Chen et al., 2022) to transform the color image into 5-dimensional feature vectors in CIELAB color space and XY coordinates and then construct distance metric standards for the 5-dimensional feature vectors to perform local clustering of image pixels. The dark part of the image is converted into a super pixel image array, and the adjacent pixels in the super pixel array are released regularly so that there is a similarity relationship between them; thus, the shortest distance between the similarity degrees can be calculated as follows:

$$f_{hrp}\left(q,w\right)=\underset{q_{1}=q,q_{2},\ldots ,q_{i}=w}{\min}\sum_{n=1}^{i-1}f_{sqq}\left(q_{n},q_{n+1}\right)$$ (6)

Where *hrp* stands for the shortest similarity distance from *q* to *w*, *f*_*hrp*_ represents the number of similarity systems between dark fragments *q* and *w* of super pixels, and *f*_*sqq*_ represents the Euclidean distance of colors in a super pixel abstract space. According to the calculation results, the block area of the dark part image can be obtained as follows:

$$\mathrm{Area}\left(q\right)=\sum_{n=1}^{l}\exp\left[-\frac{f_{hrp}^{2}\left(q,q_{n}\right)}{2S_{vat}}\right]=\sum_{n=1}^{l}D\left(q,q_{n}\right)$$ (7)

The fusion value of the contour perimeter and dark pixel fragment area is calculated as follows:

$$BndCon\left(q\right)=\frac{Len_{bnd}\left(q\right)}{\sqrt{\mathrm{Area}\left(q\right)}}$$ (8)

**FIGURE 3:**
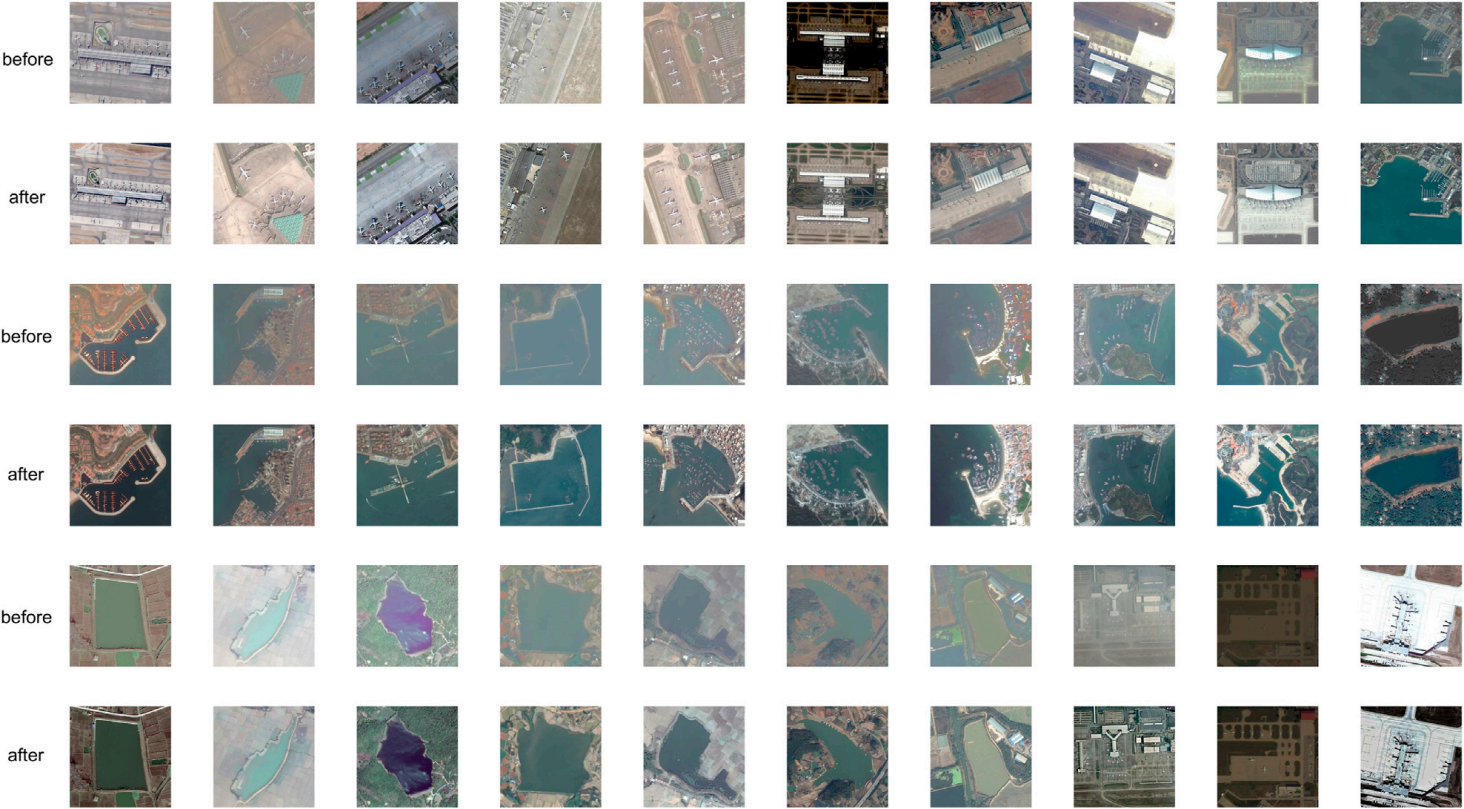
Comparison of light and dark scenes before and after separation.

On normal conditions, the boundary shading values of the target pixel and the background pixel are different. The boundary shading values of the target pixel tend to be closer to 0, while the boundary shading values of the background pixel tend to be closer to 1. Therefore, a self-defined segmentation value can be used to achieve the segmentation of light and dark scenes and obtain the saliency region (Sun et al., 2022). When the impulse noise is very large, the TMF suppression effect is very outstanding. Figure 3 is the effect diagram after the separation of light and dark scenes. The algorithm separates the target white scene from the background dark scene and effectively enhances the edge. After the above calculation, the image can basically reach the recognition standard.

### Principles of the YOLO Algorithm

The first step of traditional target detection is to extract features, such as the LBP feature and HOG feature. Secondly, object model can be trained by SVM approach, then matches the model with the target. YOLO uses the idea of regression to integrate target region prediction and target category prediction into a neural network model. In this method, a neural network is used to unify candidate frame extraction, feature extraction, target classification and target location to achieve end-to-end target detection.

**FIGURE 4:**
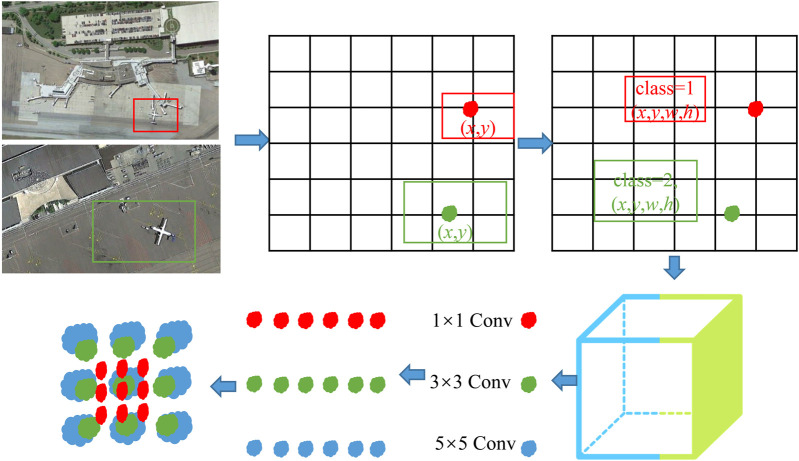
Detection principle of the YOLO model.

Biomimetic sensing images may contain multiple targets or categories of targets, so it is necessary to judge multiple categories of each prediction frame. The complete detection process of YOLO is shown in Figure 4. The specific detection process is as follows:

1) The image is divided into *S* × *S* grids. Each grid is given B prediction frames. Each prediction frame contains 5-dimensional information, namely, (*x*, *y*, *w*, *h*, *c*), where (*x*, *y*) is the predicting boundary centre relative to the offset of cell boundaries, (*w*, *h*) is the boundary of the width and height relative to the proportion of the whole image, and *c* is an incredible value, that is, the confidence that the target is included in the prediction boundary, as shown in Formula (9). Here, *Pr*(*o*) indicates whether the target exists in the cell corresponding to the prediction frame, one indicates existence, and 0 indicates nonexistence. IoU is the intersection ratio between the prediction box and the real value.

c=Pr(o)×IoU (9)

2) CNN extract characteristics and prediction of each grid are presented when an object of class c has the conditional probability *Pr* (*c*|*o*), and then the probability of each category in the network is obtained. The probability of a certain class is multiplied by the corresponding confidence, and the confidence value of the class is obtained, as shown in Formula (10):

c=Pr(c|o)×Pr(o)×IoU=Pr(c)×IoU (10)

3) The filter frame is suppressed according to the non-maximum value, then output the final judgement result. To optimize the model, YOLO uses the *S* × *S* × (*B* × 5 + *c*) dimension vector and mean square error of the image truth value as the parameters of the loss function. However, since there are no target objects in many meshes, different scale factors are set to balance the predicted boundaries regardless of the existence of targets when designing the loss function of YOLO, and the loss factors of boundary boxes need to be distinguished.

For the loss coefficient of category judgement, for example, the loss weight of the boundary box is set to be 10 times the loss coefficient of category judgement. The above design gives the loss coefficient of the boundary box a higher weight. The form of the loss function is shown in Formula (11).

$$l=\alpha \sum_{i=0}^{S^{2}}\sum_{j=0}^{B}\left[{\left(x_{i}-\bar{x}\right)}^{2}+{\left(y_{i}-\bar{y}\right)}^{2}+{\left(x_{j}-\bar{x}\right)}^{2}+{\left(y_{j}-\bar{y}\right)}^{2}\right]$$ (11)

### Addition of a Vertical Grid to Improve the YOLO Network

In the YOLO detection method, images are divided into *S* × *S* grids, that is, horizontal and vertical detection weights are the same. However, the length-to-width ratio of the target in the enlarged image obtained after biomimetic remote sensing image correction cannot accurately reflect its true value. The nonlinear target deformation and different deformation density in the same direction showing a phenomenon of imbalance between the upper and lower parts.

**FIGURE 5:**
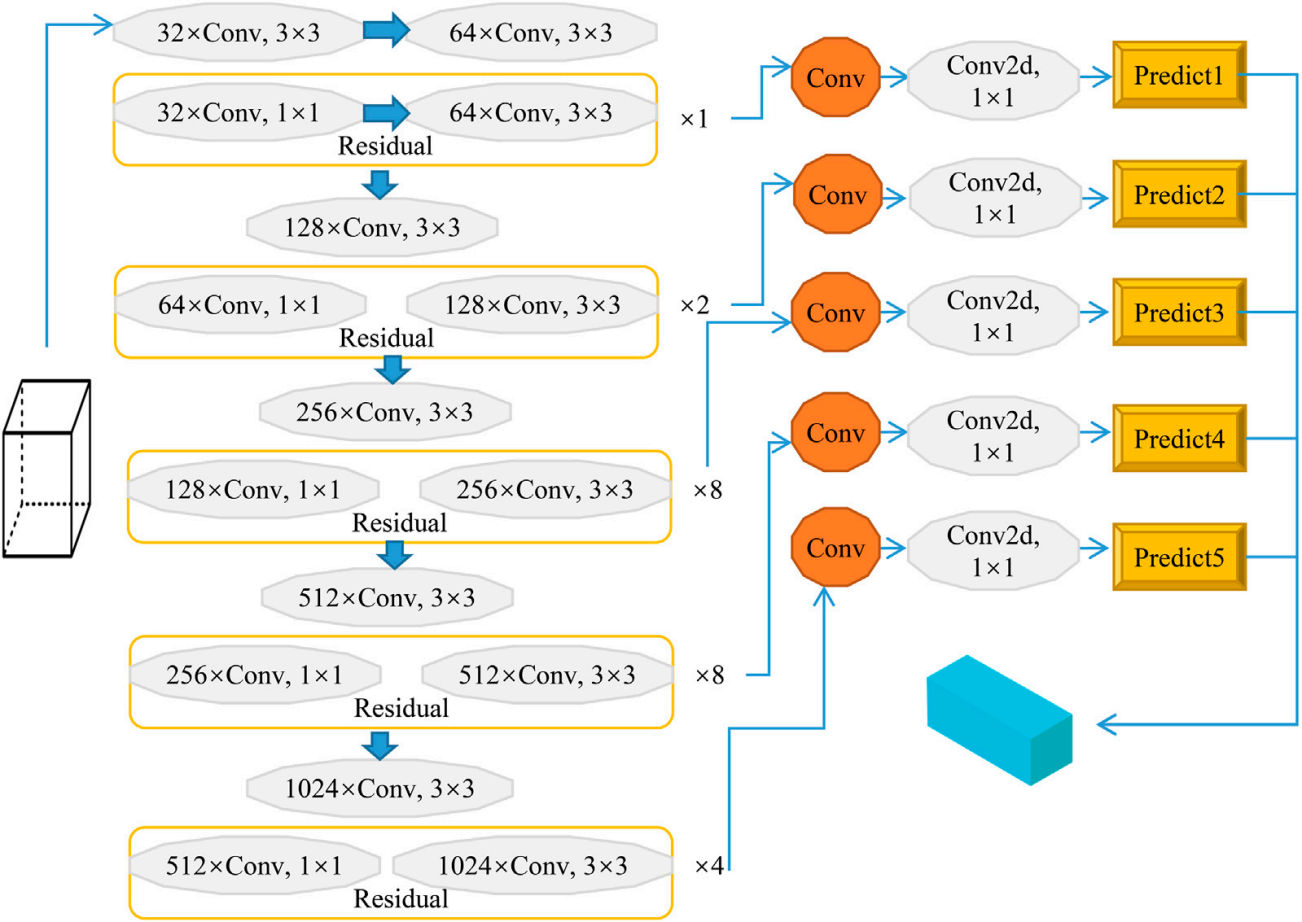
Improved YOLO model for high spatial resolution biomimetic remote sensing target detection.

Taking the spherical center of the curved zoom of the bionic human eye as the origin of the coordinate system into consideration, if we want to use the linear model to locate the target, what need to obtain the positions of two or more pixels of the same target to determine the three-dimensional world coordinates of the target point in space. The camera passes through the lens imaging system, and its projection can convert the three-dimensional scene into the two-dimensional plane of the camera through imaging transformation. To solve this problem, the predicted frames in the vertical axis direction are altered. In this paper, the vertical number is doubled without changing the horizontal number; that is, the grid number is changed from S × S to S × 2S, and it is increased at the end of the YOLO network structure. This network structure includes 20 convolutional layers and five maximum set layers and generates the improved YOLO network, as shown in Figure 5, to meet the needs of high spatial resolution remote sensing image detection.

## Experiments and Results

### Dataset Pre-processing

The application scenario of the experiment in this paper is target detection from the perspective of high-resolution zoom sensing. Therefore, the dataset used must have the characteristics of zoom sensing images, and the common characteristics are as follows: 1) This is one of the most distinctive characteristics of biomimetic remote sensing images, which means the image is acquired from a high-altitude view, presenting a picture effect of the bird’s eye view. Therefore, the detector developed by conventional data training cannot achieve ideal results. 2) Scale diversity. As the flight height is not fixed when acquiring data, the shooting height of remote sensing images is uneven, which results in different sizes of ground targets, furthermore even similar targets will show different sizes. 3) Target complexity. This is the difficulty of zoom target detection training. Since the most targets in this scene are small targets composed of dozens of or even several pixels, feature information is scarce. In addition, remote sensing images contain too many targets, as well as different target states and directions, resulting in very complicated and difficult training. 4) Background interference. Zoom sensing images usually cover a coverage area of several square kilometers, with a large field of vision. Therefore, the background often contains rich information, such as plain green land, mountains, rivers and road courses, with strong interference.

To make the data meet the above characteristics, the collection of datasets needs a special aerial zoom sensing image database. Commonly used open datasets include aerial image dataset (AIDDataset), DOTA, UCAS-AOD, NWPUBHR-10, etc. The AIDDataset used in this paper is a remote sensing image dataset that contains scene images of 30 categories, of which each category has approximately 220–420 images, and the overall total is 10000 images, of which each pixel size is approximately 600 × 600. The dataset, published by Huazhong University of Science and Technology and Wuhan University in 2017, contains a wide range of images under different imaging conditions, with different sizes of the ground and objects in the images.

**FIGURE 6:**
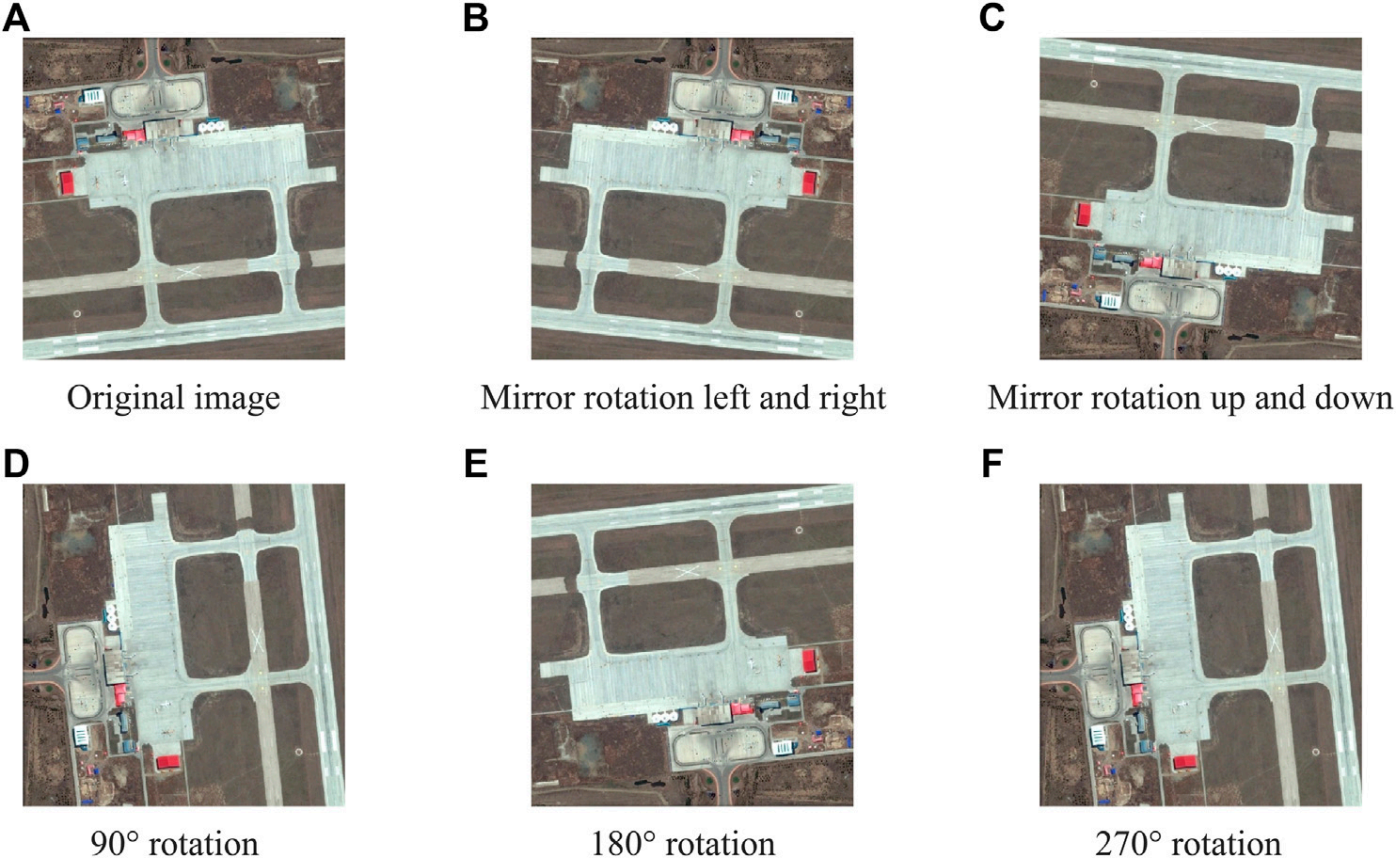
Dataset enhancement example.

Preliminary processing of the dataset is as follows: First, the TIF format data of zoom sensing images after multispectral and panchromatic data fusion are converted to PNG format, which is commonly used in network models. Then, the training data and test data suitable for network model training are screened. The dataset postprocessing includes five stages: data annotation, mask generation, sample cutting, down sampling and data enhancement. Because there is too much texture information in high-resolution zoom sensing images, it will affect the judgement of the network model and reduce the segmentation accuracy. Therefore, the sample size of 600 × 600 was reduced to 256 × 256. To enrich the image training set, better extract the training features, and generalize the model (to prevent overfitting), image block rotation, distortion, increase of noise and other ways to enhance the image were utilized. Specifically, the original image is flipped 90°, 180°, and 270°, and it is mirrored horizontally and vertically to generate another five pieces of data. The original data are added, and the amount of data increases by 6 times. The same operation is performed on the label, as shown in Figure 6. Through the above operation, the new training set contains 12000 pieces 600 × 600 sub images. To evaluate the training effect of the proposed network model on different datasets, the data set used for the test is divided into test set 1 (20000 pieces, 256 × 256), testing set 2 (20000 pieces, 512 × 512) and testing set 3 (20000 pieces, 600 × 600).

### Algorithm Setting and Detection Speed

The training and testing of the network model are carried out in the server of the laboratory. The configuration of the computer is Windows 10 system, the CPU is Intel core5-7200u, the GPU is NVIDIA GeForce GTX940, and the memory is 8G. Cuda10.2, python3.6, and the corresponding extensions are installed.

Torch can be applied to a wide range of algorithms for machine learning. Compared to Caffe, the interface is easier to use, and the bottom layer is the C/CUDA execution program. PyTorch, a version of Torch, is a Python-first deep learning framework that uses a powerful GPU such as Numpy to speed up tensor calculations. PyTorch’s interface is flexible and easy-using, with more excellent performance than other frameworks. In summary, considering the environment required for this experiment, the PyTorch framework is adopted in this paper as the platform for YOLO zoom sensing target detection by comparing the characteristics, performance requirements, deployment conditions and ease of use of the network model.

Limited by the laboratory computer configuration, the batch size was 2, in which the default optimal value of the YOLOv5 algorithm was adopted for momentum. Some parameters during training were selected, as shown in Table 1, and experiments were carried out after parameter setting was completed.

**TABLE 1:** Selection of key parameters.

Batch size	Epoch number	Momentum	Decay	Learning rate
2	300	0.937	0.0005	0.01

**FIGURE 7:**
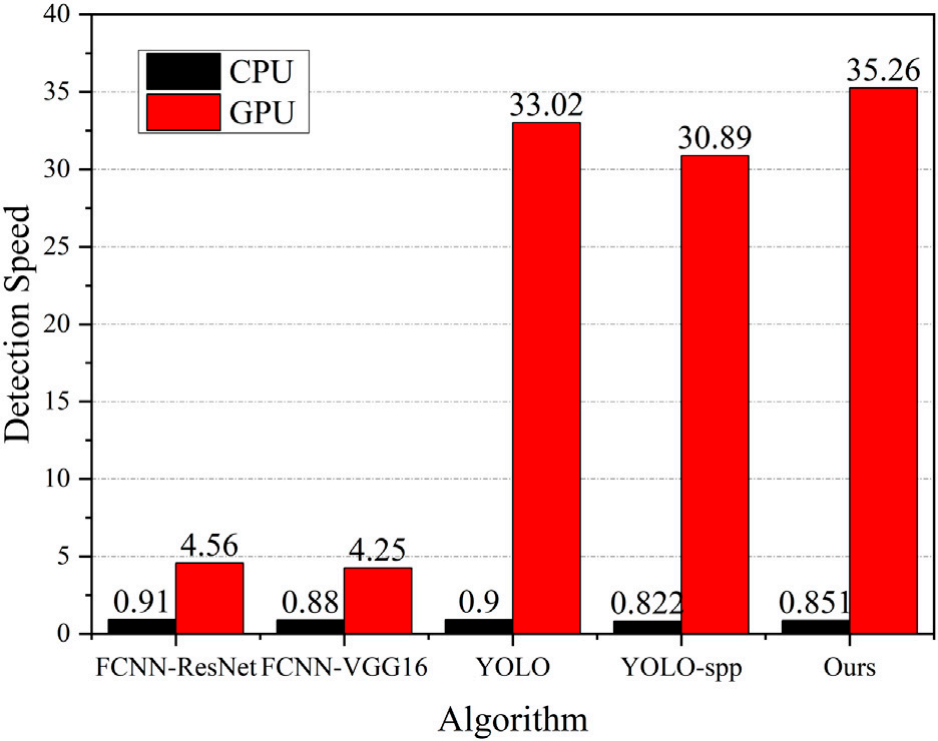
Comparison of detection speed.

The improved algorithm and other algorithms were tested on a CPU and GPU, respectively. The statistical results are shown in Figure 7. The detection speed of the algorithm is calculated according to the average number of test images per second. The more pictures processed, the faster the detection speed of the algorithm.

No CPU can meet the real-time requirements of zoom sensing detection. The speed of using YOLO or the proposed algorithm on a GPU is more than 30 pieces per second, which far meets the detection speed requirements. The current mainstream detection method, Faster-RCNN, has a higher accuracy rate, but the detection speed is less than five frames per second. It embodies the excellent performance of YOLO and the improved YOLO algorithm in zoom sensing real-time detection.

**FIGURE 8:**
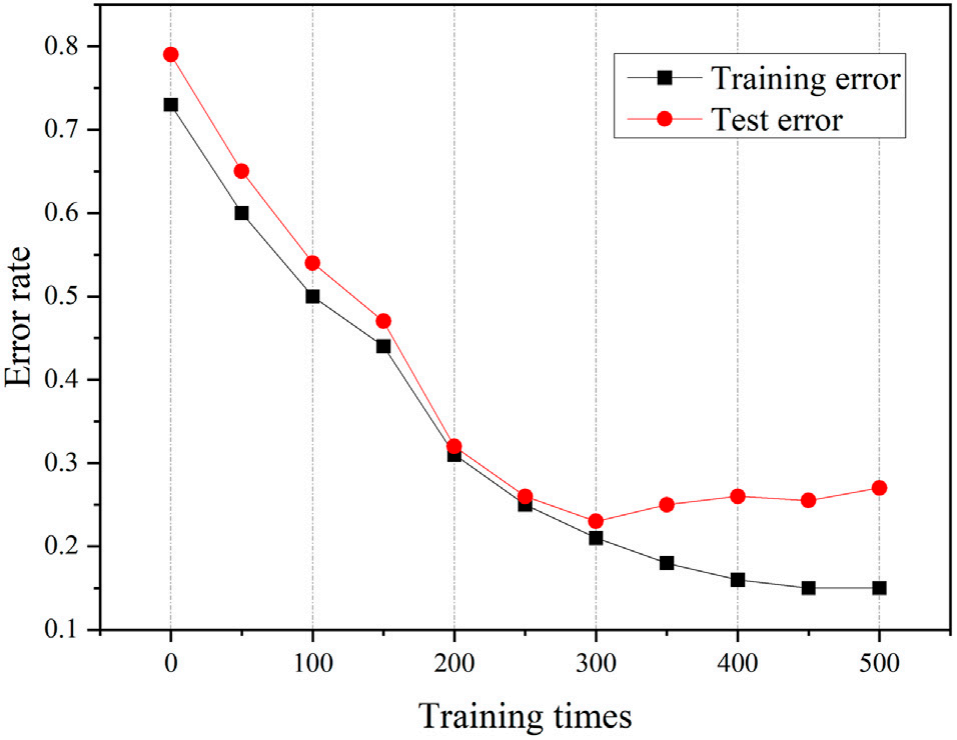
Variation curves of training error and test error over time.

**FIGURE 9:**
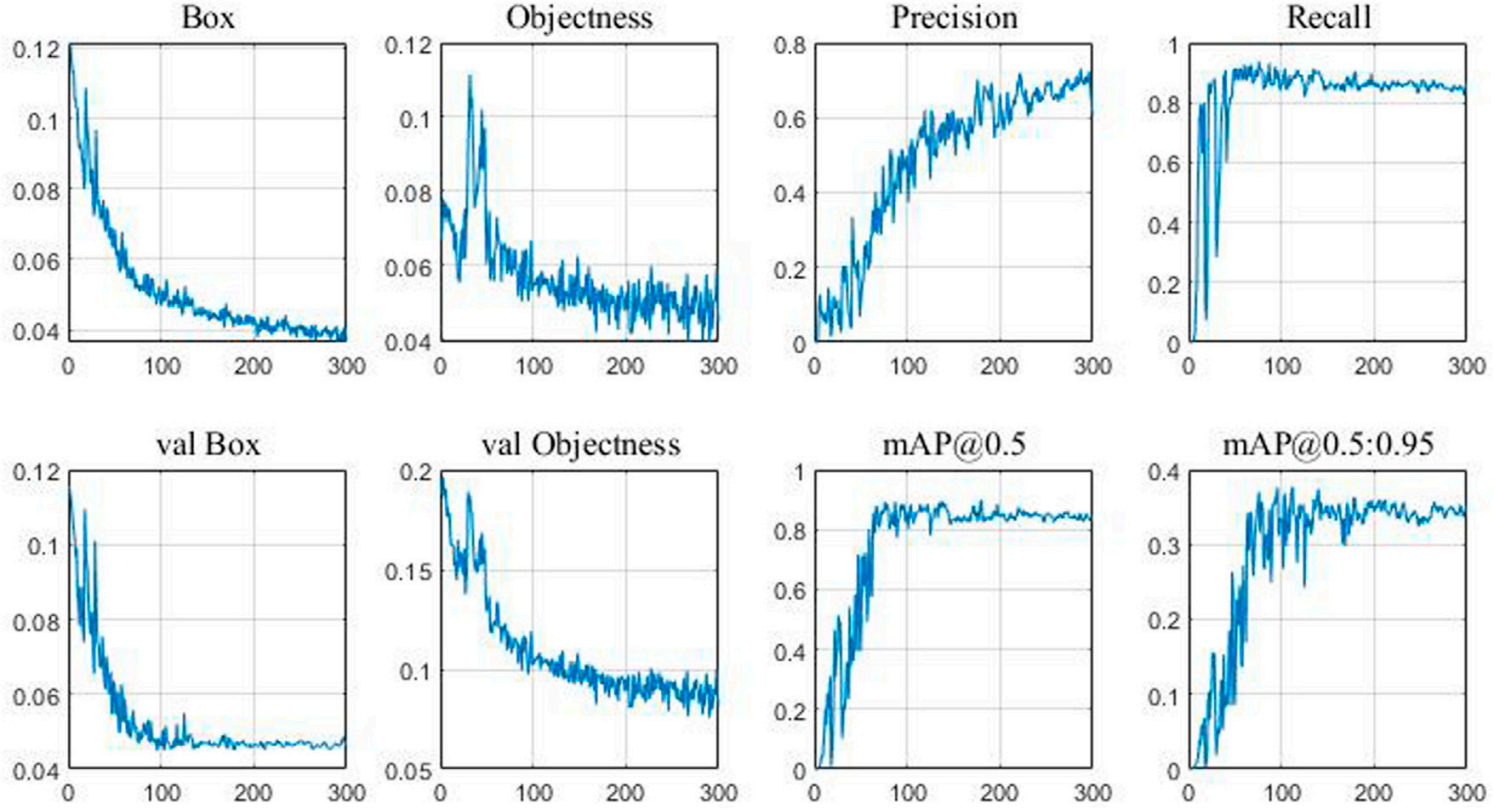
Visualization of training results.

The curve of training error over time is shown in Figure 8. It can be seen from the figure that the training error continuously decreases with the progress of training and finally reaches the lowest error rate of 21%. However, the test error reaches the lowest error rate of 23% when the training reaches 300 times, and then the error rate increases. Therefore, the optimal classification model finally obtained is the model trained 300 times. The model parameters obtained at the 300th training session are shown in Figure 9. YOLOv5 uses GIOU loss as the loss of bounding box. Box is the mean value of GIOU loss function. The smaller the value, the more accurate the box is. Objectness is speculated as the mean value of target detection loss. The smaller the target, the more accurate the target detection is. Classification is the mean value of classification loss. The smaller the classification, the more accurate the classification is. Precision indicates that the correct positive class is found. Recall means the true positive accuracy, that is, how many positive samples have been found. From the perspective of recall, the number of real classifiers is described, that is, how many real classifiers are recalled. Val BOX is the verification set bounding box. Val Objectness is the mean value of target detection loss in the verification set. Val Classification is the mean value of the classification loss of the verification set. mAP is the area surrounded by precision and recall as two axes, m represents the average, and the number after at represents the threshold for judging IoU as positive and negative samples.

### Comparison of Accuracy

**FIGURE 10:**
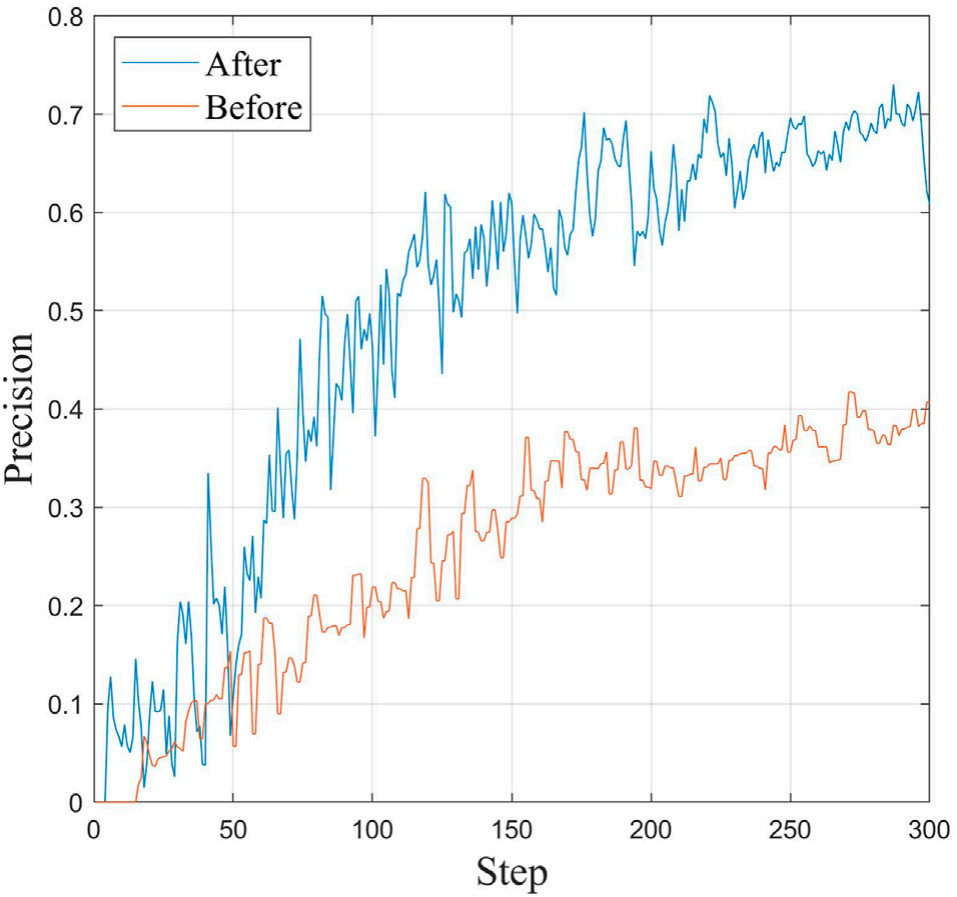
The improved YOLO algorithm and the change in accuracy with training times before improvement.

The accuracy of target detection can be indirectly reflected by the detection error rate. The lower the error rate is, the more reliable the detection accuracy of the corresponding model is, as shown in Figure 10. The following conclusions can be drawn.

1) The zoom sensing image is directly detected without preprocessing, the detection effect is very unsatisfactory, and it has lost practical value. In this paper, the YOLO expansion diagram is used for detection, and the error rate is approximately 35%. Because the detected target is in a “short and thick” state, traditional detectors cannot adapt to it. Using the algorithm in this paper, by increasing the number of longitudinal prediction frames to adapt to short and thick targets, the error rate is controlled at approximately 30%, and the performance is far better than that of YOLO.
2) Compared with Faster-RCNN and other detectors, the error rate of Faster-RCNN is less than 30%, which is the detector with the best accuracy. The error rate of the proposed algorithm is slightly higher, approximately 3%, but considering the huge advantage of the detection speed of the proposed algorithm, it is more practical in high spatial resolution zoom sensing image detection.

The phenomenon of misclassification and missing classification is obvious in the traditional Faster-CNN method, and the edge details are rough and messy, so it cannot effectively identify the real object category in the shaded area, and it cannot accurately classify small targets such as aircraft. This is because traditional classification methods are restricted by many factors, such as the image segmentation scale and classifier performance. The deep learning method FasterCNN-VGG16 has fewer errors and omissions, but it is not effective in distinguishing low vegetation and trees and processing details such as building edges and vehicles. This is due to the phenomenon of large differences within the class of high-resolution zoom sensing images, i.e., the same object with different spectra or foreign objects with the same spectrum. In addition, it can be seen from the classification results of the two groups of experiments that the classification effect of existing deep learning methods is not stable. The classification effect of the proposed method is the best. After the TMP model, the light and dark scenes are separated, the edges are smooth and accurate, and the details of ground objects are reflected more comprehensively and truly.

YOLO, YOLO-spp and the improved YOLO network were trained, and the detection performance of each model was tested on the test set. To fully demonstrate the performance of the improved algorithm, Faster-RCNN and other networks are added in the experiment for comparative analysis. The comparison of detection results is shown in Table 2.

**TABLE 2:** Test results of different methods.

	mAP	Recall	Avg time
FCNN(ResNet101)	0.862	0.931	252.21
FCNN(VGG16)	0.874	0.922	164.16
YOLO	0.767	0.959	28.67
YOLO-spp	0.822	0.905	25.23
Our	0.851	0.972	29.5

As seen from the data in the table, the average accuracy of the improved YOLO model for all targets on the test set is 85.1%, and the recall rate is 97.2%, which is increased by 8.4 and 1.3%, respectively, compared with YOLO. The accuracy of all single targets was improved. The mAP values of YOLO and other detection algorithms in its series, such as YOLO-spp, are 76.7 and 82.2%, respectively, and the recall rates are 95.9 and 90.5%, both lower than that of the improved YOLO model. For the detection results of a single target, as shown in Table 3, the map value of any other algorithm, whether in parking, port, storage tank or airplane, is significantly less than the optimized Yolo algorithm used in this paper, which can prove that this algorithm has a good recognition rate in zoom sensors images.

**TABLE 3:** Detection results of a single target by different methods.

Parking	Port	Storage tank	Airplane
0.468	0.731	0.893	0.901
0.423	0.732	0.858	0.908
0.439	0.657	0.765	0.825
0.503	0.692	0.906	0.916
0.876	0.852	0.901	0.919

### Angle Error Test

**FIGURE 11:**
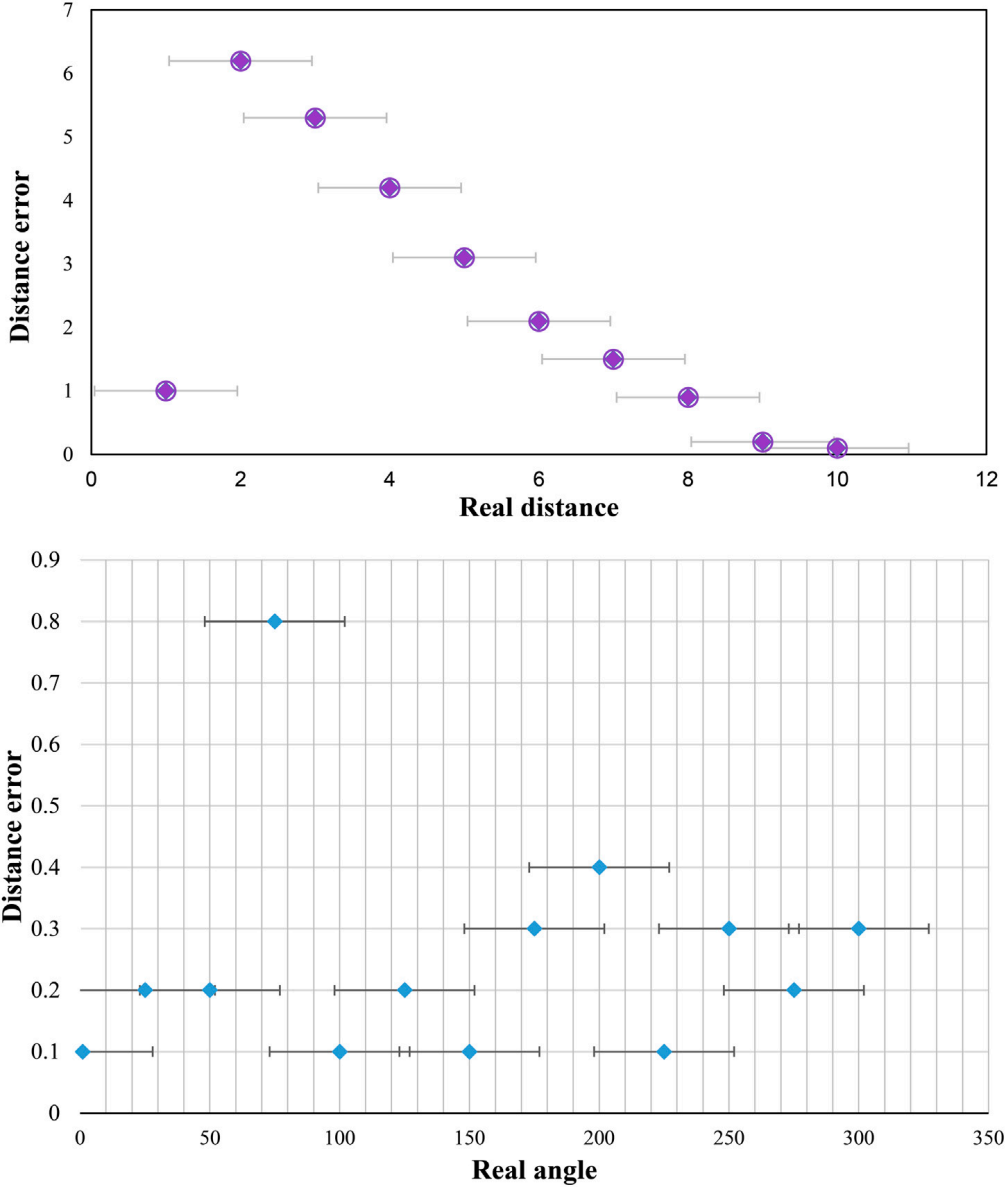
Static distance and angle error.

Figure 11 shows the angle error of the zoom sensing image obtained by the drone. Figure 11 shows that the mean error and standard deviation of targets at different angles are basically the same, which indicates that the improved YOLO model has good detection accuracy.

**FIGURE 12:**
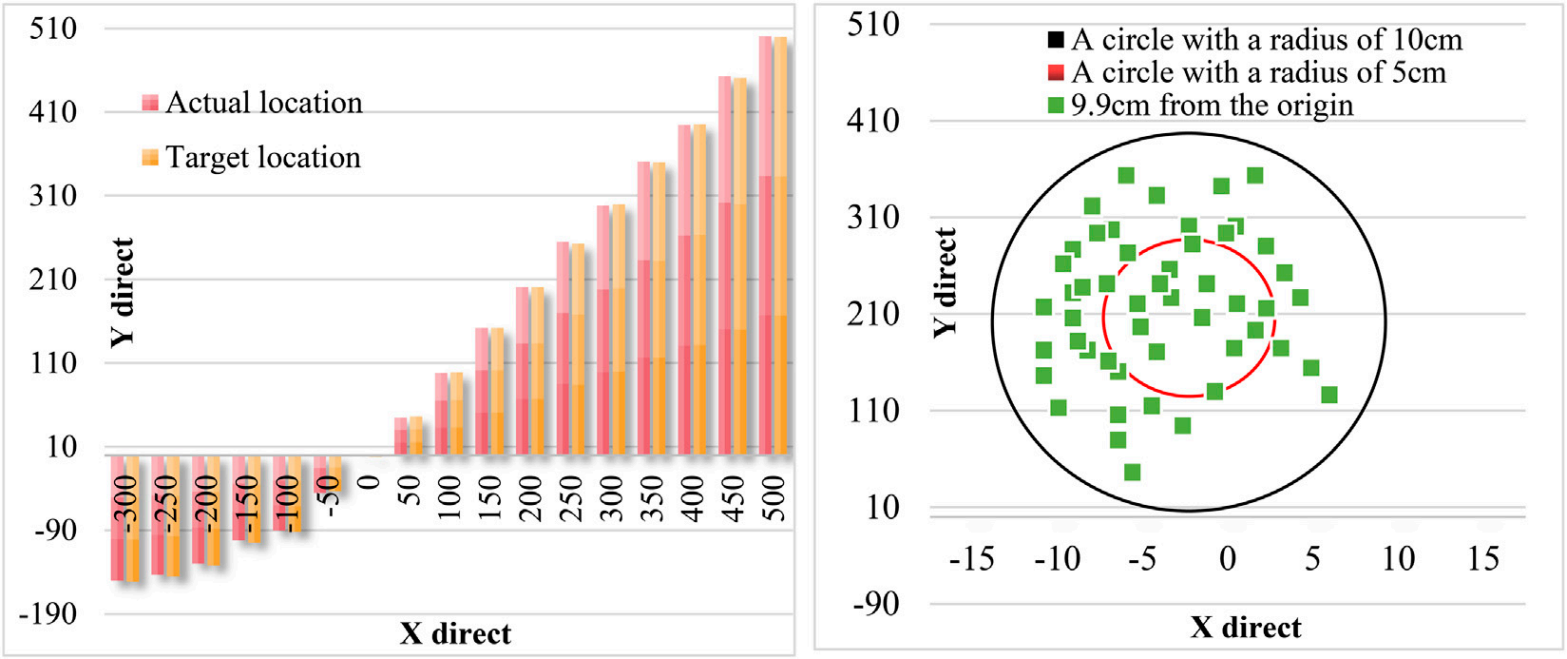
Position comparison diagram and static error diagram.

Figure 12 is the comparison diagram and static error diagram of the observed actual position and the position obtained by the visual system. As seen from the figure, the maximum static distance error of the visual system is 9.9 cm, which is not much different from the real-time detection error.

Experimental results show that the improved YOLO algorithm not only has good comprehensive performance in terms of detection speed and detection accuracy but can also meet the detection requirements when adapting to target differences at different angles in zoom sensing target detection.

## Conclusion

In this paper, the resolution of bionic sensing images and target detection accuracy are improved by optimizing some parameters of texture features in the light and dark areas of zoom images. A target detection method based on the improved YOLO algorithm is proposed. Experimental results show that the average precision of this method for all targets images on the test set in the GPU environment is 85.10%. Finally, compared with traditional YOLO algorithm, the precision and recall rate are increased by 10.91 and 1.40%, respectively, and the detection speed is increased by 6.78%. In addition, the average error and standard deviation of targets at different angles are basically the same with good detection accuracy, which meets the target detection requirements of high-resolution zoom sensing images, and the accuracy of target recognition is maintained at greater than 70%.

## Data Availability

The original contributions presented in the study are included in the article/Supplementary Material, further inquiries can be directed to the corresponding author.
